# A challenging diagnosis of plasmablastic lymphoma: importance of integrating morpholgy immunohistochemistry and flow cytometry findings (case report)

**DOI:** 10.11604/pamj.2023.45.158.39896

**Published:** 2023-08-13

**Authors:** Zahra Kmira, Gereisha Ahmed, Cherif Wided, Moatamri Wided, Nfikha Zeineb, Abdessaied Nihed, Ben Youssef Yosra, Haifa Regaieg, Brahem Nejia, Khelif Abderrahim

**Affiliations:** 1 Department of Clinical Hematology, Farhat Hached University Hospital, Sousse, 4081, Tunisia,; 2 Department of Cytology, Farhat Hached University Hospital, Sousse, 4081, Tunisia,; 3 Department of Pathology, Farhat Hached University Hospital, Sousse, 4081, Tunisia

**Keywords:** Plasmablastic lymphoma, flow cytometry, immunohistochemistry, case report

## Abstract

Plasmablastic lymphoma (PBL) is a rare clinicopathological entity that still raises many diagnostic and management difficulties, particularly due to the overlap between plasmablastic lymphomas and myeloma features. We report a clinical presentation of PBL affecting bone marrow in a 43-year-old patient who was admitted for B symptoms, hepatosplenomegaly, and bicytopenia investigation. Based on these findings, acute leukemia was suspected. Bone marrow morphology immunohistochemistry and flow cytometry contributed to establishing the diagnosis of medullary PBL. The patient deteriorated and died due to septic shock. This pathology requires collaboration between clinicians, pathologists, and biologists to confirm the diagnosis early. Nevertheless, a delayed diagnosis may contribute to worsening the prognosis particularly due to advanced stage consultation. Our reported case illustrates a rare clinical presentation affecting bone marrow. In our context, a confrontation between flow cytometry and immunohistochemistry was of interest as it helped to detect the immunological features of this neoplasm.

## Introduction

Plasmablastic lymphoma (PBL) is a rare and uncommon aggressive lymphoma composed of immunoblasts or plasmablasts characterized by a CD20 negative phenotype. It constitutes a new subtype of aggressive mature B-cells neoplasm as defined by the WHO classification of lymphomas and hematological malignancies WHO 2016 [1]. Its diagnosis is difficult because its features overlap with myeloma and lymphoma. Given its rarity, there is currently no consensus on its treatment. In this report, we present the diagnosis difficulties of PBL in an HIV-negative patient.

## Patient and observation

**Patient information:** a 43-year-old male was referred to the hematology department for exploration of anemia and thrombocytopenia. His personal and family history was negative for hematologic malignancies. The patient reported two months symptoms evolution including asthenia, anorexia, progressive weight loss, inferior members swelling, and pruritus.

**Clinical finding:** the physical examination showed a pale patient with performance status at 2, a mild scleral icterus, a splenomegaly, and hepatomegaly 10 and 5 cm; respectively below the costal margin. No lymph nodes were present.

**Figure 1:**
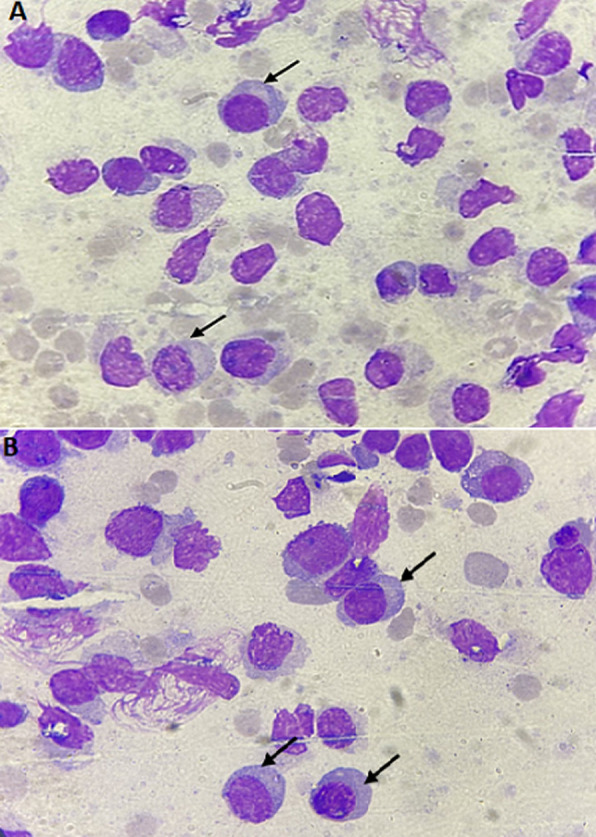
A, B) bone marrow aspirate smear examination: mature lympho-plasmacytoidic cells associated to variable size blast-like cells with thin chromatin and prominent nucleoli; may-Grünwald-Giemsa, x100 objective

**Figure 2:**
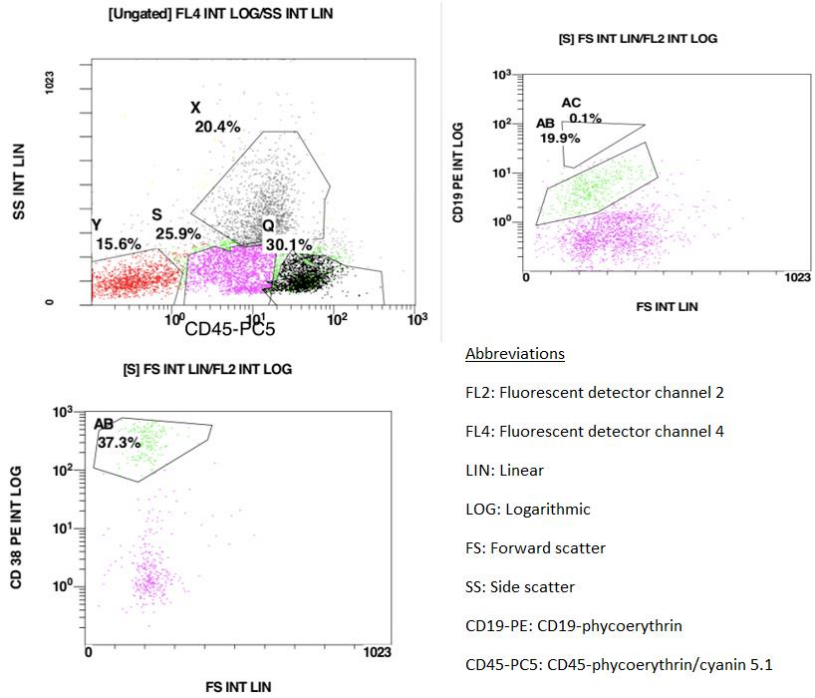
flow cytometry representation of the CD45-dim population; (gating S) presence of an immature population, (gating AB) moderately expressed CD19 with a highly expressed CD38

**Figure 3:**
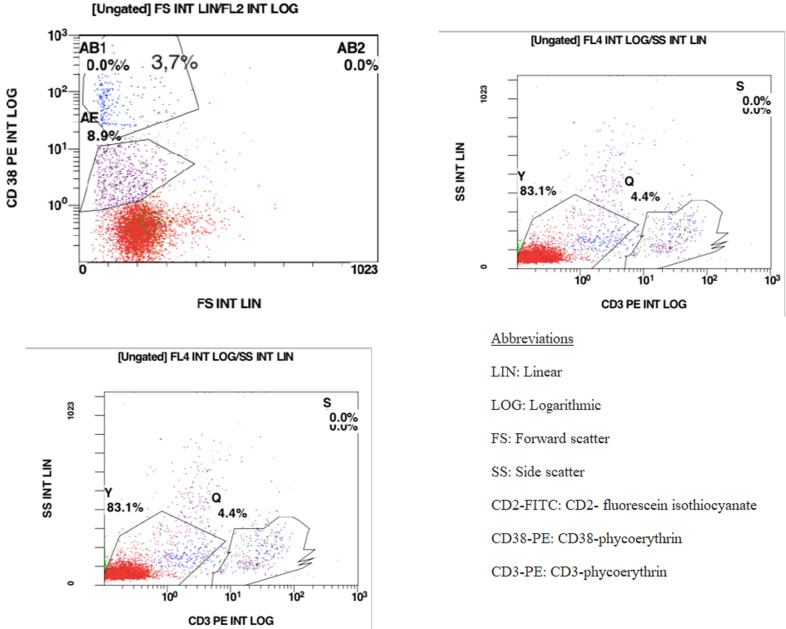
flow cytometry representation of CD2, CD3 positive lymphocytes; (AB) presence of an immature population with a highly expressed CD38 (CD2 negative, CD3 positive), (AE) normal mature lymphocyte (CD38 low CD2 positive, CD3 positive)

**Diagnosic assessment:** chest X-ray performed in the face of slight dyspnea showed a bilateral moderate pleural effusion without compression signs. CBC showed a white blood cell count 44950/mm^3^, 27% granulocytes, 28% lymphocytes, 13% monocytes, 1% eosinophils, 8% immature granulocytes, and 23% lymphomatous cells, hemoglobin 4.5g/dl platelets 30,000/mm^3^. He had impaired renal function (serum creatinine = 22.6 mg/L, MDRD= 41 mL/min/1.73m^2^), high levels of lactate dehydrogenase at 497UI/l (twice the upper limit of normal value) and negative direct Coombs test. Hepatitis B, C, and HIV serologies were negative. Based on these findings the diagnosis of acute leukemia was suspected leading to perform bone marrow aspiration showing abnormal mononuclear cell fraction (lymph-plasmocytes= 28%, blast-like cells=42%). The rest of the smear included the following percentages: 4% of immature granulocytes, 6% of neutrophils, 6% of lymphocytes, and 18% of erythroblasts. The medullary smear was hypercellular, totally infiltrated with mature lympho-plasmacytoidic cells associated to variable-size blast-like cells with thin chromatin and prominent nucleoli. The cells have an extended agranular cytoplasm with variable basophilia and the myeloperoxidase staining returned negative (Figure 1). Cytogenetic study of the bone marrow cells revealed a normal Karyotype. Flow cytometry analysis on bone marrow aspirate, performed using the following panel: IgG1, CD45, CD34, HLA-DR, CD33, CD13, CD65, CD117, CD10, CD19, CD3, CD2, CD4, CD56, Igκ, Igλ, CD38, CD22, CD20, CD29, CD58, concluded to the absence of acute leukemia markers. However, it revealed the presence of an immature population with a highly expressed CD38 and moderately expressed CD19. The fluorescence intensity was different when compared with the lymphocytes´ profile (CD38 low and CD19 high) (Figure 2 and Figure 3).

A peripheral blood flow cytometry assessment, indicated in order to explore a suspected lymphoproliferative disorder, using the following panel markers: CD19, CD5, FMC7, CD23, CD22, CD20, CD10, CD79b, Igκ, Igλ, CD2, CD38, CD103, CD11c, CD43, CD25, and CD45, showed the absence of phenotypical aberrations in the lymphocytes. Regarding the medullar infiltration with mature lympho-plasmacytoidic cells, further biological explorations were performed. The 24-hour urine protein test showed proteinuria at 31,23g/24h with a negative bacteriological urine examination culture. The serum protein electrophoresis demonstrated a beta-2-globulin fraction spike (8.8%). A complementary investigation by immunofixation demonstrated the presence of a “lambda light chain spike with a decrease of immunoglobulins.

**Figure 4:**
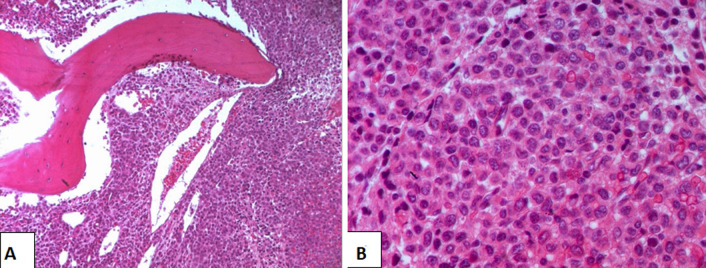
bone marrow location of a plasmablastic lymphoma; medullary spaces are infiltrated with a dense lymphomatous proliferation (hematoxylin eosin x100); tumor cells are medium to large sized and show diffuse plasmacytic differentiation with abundant cytoplasm and eccentric nuclei (hematoxylin eosin x400)

**Figure 5:**
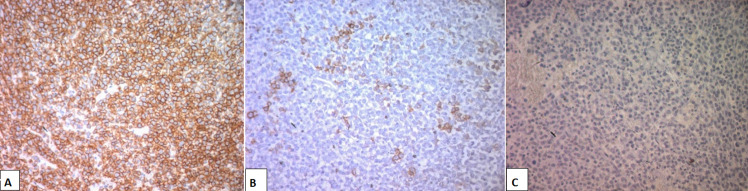
immunohistochemistry of the plasmablastic cells (x200); (A) cells are strongly positive for CD138; (B) cells are focally positive for EMA; (C) cells are negative for CD20

**Diagnosis:** regarding the difficulty in confirm the diagnosis, a bone marrow biopsy was performed showing a morphological aspect and an immunohistochemistry profile compatible with a PBL. In fact, the bone marrow specimens revealed an extensive infiltration with medium to large-size tumor cells with moderately abundant eosinophilic cytoplasm and a hyperchromatic exenterated nucleus forming a plasmacytic aspect. Nuclear atypia was observed, and mitosis figures were frequent. A fibrous background was observed with reactional lymphocytes (Figure 4). Immunohistochemistry showed a diffuse positivity of the tumor cells for CD138, focal expression of EMA (Figure 5), and was negative for CD45, CD79a, CD3, CD30, CD5, CD10, MuM1, Bcl6, and Cyclin D1.

**Therapeutic interventions:** the patient was initially stabilized by hyperhydration and red blood cell transfusions. After the diagnostic confirmation, he received dexamethasone at the dose of 40 mg/day for 4 days with good clinical evolution and improvement in renal function.

**Follow-up and outcome of interventions:** waiting for the approval of chemotherapy, the patient consulted a week after hospital discharge for fever and deterioration of his general state causing his death by septic shock. No autopsy was made.

**Informed consent:** the patient gave informed consent.

## Discussion

Plasmablastic lymphoma, described initially as a malignant diffuse B cells lymphoma characterized by its aggressive and relapsing clinical presentation, was considered in the revised 2008 WHO classification of lymphoid neoplasms as a distinct entity rather than a part of diffuse B cells lymphomas [1]. It is a clinicopathological entity that still raises many diagnostic and management difficulties particularly due to the overlap between plasmablastic morphology lymphomas and myeloma. It is a rare entity that constitutes less than 3% of non-Hodgkin lymphomas [1]. Plasmablastic lymphoma was first described as an HIV-associated neoplasm. However, many reports concluded that PBL does not exclusively occur in an immunodeficiency context such as organ transplantation or immunosuppressive treatments but may also affect immunocompetent individuals. The epidemiological particularities of this neoplasm are generally dependent on the clinical case presentation. Patients´ mean age is generally characterized by its bimodal distribution from 40-49 years in HIV-positive individuals to 67 years in HIV-negative cases. There is a strong male predominance [2]. Plasmablastic lymphoma is characterized by a frequent involvement of the oral cavity particularly in HIV-positive patients as initially described by Jacques Delécluse. Following this first report, many authors tend to affirm that the most implicated manifestation sites are the oral cavity followed by the gastrointestinal tract, lymph nodes, and skin in both HIV-positive and HIV-negative patients [3]. Other less common sites include the central nervous system, mediastinum, lungs, and liver. Most cases tend to present with rapidly progressive symptoms and an advanced disease stage (Ann Arbor stage III or IV) [2] with elevated LDH values and B symptoms [3] as presented by our patient. Plasmablastic lymphoma is considered a high-grade neoplasm.

It is generally composed of immunoblasts or plasmablast-like cells with varying degrees of plasmacytic differentiation. Immunoblastic cells are usually characterized by a moderate to abundant cytoplasm, vesicular chromatin, and most of all a prominent central nucleolus. This pattern is generally observed in HIV-positive individuals and is associated with an oral cavity location [4]. In contrast, in HIV-negative cases, PBL is characterized by a plasmacytic differentiation and tends to occur more frequently in other extranodal sites as well as in lymph nodes [4]. Our patient pathological features were similar to the immunocompetent individuals´ pattern. Immunohistochemistry is of great interest as PBL tumor cells exhibit a terminally differentiated B cell immunological profile and express a plasma cell-like antigenic pattern. These features could also be assessed by flow cytometry. The combined use of flow cytometry and immunohistochemistry can contribute to better diagnostic sensitivity [5]. Plasmacytic differentiation markers CD138 (member of the transmembrane heparin sulfate proteoglycan family), CD38 (cyclic adenosine diphosphate ribose hydrolase), MUM1/IRF4 (multiple myeloma oncogene 1/interferon regulatory factor 4) are generally reported positive. Epithelial membrane antigen (EMA), CD79a, and CD30 staining returns frequently positive contrary to pan B cell markers, such as CD20 and PAX-5, and CD19 whose expression is generally negative like our case [6].

A study published in 2017 by Han *et al*. reviewing 60 cases of Chinese PBL patients reported the positivity of CD138, CD38, and MUM1 in more than 80% of the cases. Meanwhile, CD20 expression was only found in 22% of the studied cases. A study published by Dorwal *et al*. reported that leucocyte common antigen CD45 is expressed in around 41% of extra-oral PBL cases opposing the studies reporting that CD45 expression is lost in the neoplastic cells [7]. Cytoplasmic immunoglobulins are generally expressed most commonly in IgG with either kappa or lambda light chain [6]. In some cases, T cell markers (CD2 and CD4) staining could return positive [2]. In situ, hybridization for EBER is commonly positive. Due to the history of immunodeficiency and the presence of EBV infection; positivity for EBER is often described in HIV-positive as well as post-transplant cases and may help establish the diagnosis [2,3]. In our case, immunohistochemistry showed a diffuse CD138 positivity, with focal EMA expression. Based on the acute leukemia suspicion, a flow cytometry assessment was indicated. Despite our panel´s limitations, we noted the presence of an immature population (gating S) which moderately expressed CD19 with a highly expressed CD38 (gating AB) (Figure 2). When we tried assessing the CD2, CD3 positive lymphocytes, we noted the presence of an immature population with a highly expressed CD38 (AB) (CD2 negative, CD3 positive) distinguished from normal mature lymphocyte (AE) (CD38 low CD2 positive, CD3 positive) (Figure 3).

Plasmablastic lymphoma is considered as a real therapeutic challenge as it is an aggressive malignancy with a poor prognosis. There is an extensive range of treatments possibly administrated to patients with PBL from radiotherapy in the context of patients with localized disease and focal lesions, to different chemotherapy protocols in patients with a disseminated disease. Chemotherapy protocols including CHOP (cyclophosphamide, doxorubicin, vincristine, and prednisone), and CHOP-like regimens have been used in most of the patients reported to date and actually, are considered inadequate due to the highly aggressive nature of this entity. Other regimens such as dose-adjusted EPOCH or cyclophosphamide, vincristine, doxorubicin, high-dose methotrexate (CODOX-M)/ alternating with ifosfamide, etoposide, and high-dose cytarabine (IVAC) or hyper fractionated cyclophosphamide, vincristine, doxorubicin, and dexamethasone alternating with methotrexate and cytarabine (hyper-CVAD) can be considered as treatment options for this highly aggressive lymphoma [8]. As PBL cells display a degree of plasmacytic differentiation, therapeutic agents that are used in myeloma treatment have also been used to treat PBL with variable degrees of success. Anti-CD30 antibodies have been reported to be associated with a rapid response and reduction of tumor size [9]. The prognosis of this neoplasm is poor characterized by an overall median survival of 8 months [4], especially as patients present at an advanced stage [10]. No clear difference has been reported between HIV-positive or HIV-negative patients [4,5].

## Conclusion

Plasmablastic lymphoma remains a challenge in both diagnosis and treatment aspects. Therefore, it requires collaboration between clinicians, pathologists, and biologists. Our reported case illustrates a rare clinical presentation affecting bone marrow. In our context, a confrontation between flow cytometry and immunohistochemistry was of interest as it helped to detect the immunological features of this neoplasm despite our panel limitations.
